# Giant staghorn stone causing inferior vena cava compression: a novel case report

**DOI:** 10.1097/MS9.0000000000000680

**Published:** 2023-05-03

**Authors:** Mohammad A. Alghafees, Saleha Abdul Rab, Hiba M. Raheel, Belal N. Sabbah, Ahmed E. Maklad, Mazin I. El Sarrag, Ahmed E. Abouelkhair, Abdulaziz Aljurayyad, Tariq Alotaibi, Mohammed T. Alotaibi, Mohammad Alomar

**Affiliations:** a College of Medicine, King Saud Bin Abdulaziz University for Health Sciences; b College of Medicine, Alfaisal University; c Department of Urology, King Saud University Medical City; d Department of Urology, King Faisal Specialist Hospital and Research Centre, Riyadh, Saudi Arabia

**Keywords:** case report, giant staghorn stone, hepatosplenomegaly, inferior vena cava compression, percutaneous nephrolithotomy (PCNL), polycythemia vera

## Abstract

Staghorn calculi (SC) are defined as large kidney stones that fill the renal pelvis and at least one renal calyx. They represent 10–20% of all renal stones in developing countries and require prompt diagnosis and management. Massive SC (over 5 cm) are treated exclusively via open surgery, despite percutaneous nephrolithotomy (PCNL) being the gold standard treatment for large stones. Descriptions of PCNL for massive SC are very limited in the literature.

**Case Presentation:**

We report a case of a 63-year-old male who presented with chronic abdominal pain, hepatosplenomegaly, and normal renal function. He was later diagnosed with polycythemia vera. Computed tomography of the abdomen revealed massive, bilateral staghorn stones measuring 7.3×5.5 cm and 1.8×4.5 cm on the right and left, respectively. Additionally, the right stone was found to be compressing the inferior vena cava (IVC). The patient was promptly scheduled for right-sided PCNL and the target of 80% stone fragmentation was successfully attained.

**Discussion:**

We present the first case of a stone of such size in the Middle East, as well as the first known case of a renal stone compressing the IVC. Unlike previous reports, the stone was successfully fragmented via PCNL – a procedure that has not been described for stones of such size.

**Conclusion:**

This report highlights that ultrasound-guided PNCL without any other intervention is sufficient for the successful treatment of giant SC. Greater research is needed on the potential utility of using ultrasound-guided PCNL for the fragmentation of stones sized over 5 cm.

## Introduction

Highlights

- Staghorn calculi (SC) are large kidney stones that fill the renal pelvis and at least one renal calyx.
- Due to their significant morbidity and mortality, prompt treatment and diagnosis of SC is vital.
- We report the first case of a staghorn stone found to be compressing the inferior vena cava.
- Current guidelines recommend the use of open, laparoscopic, or robotic surgery for the treatment of very large or bilateral SC.
- Ultrasound-guided percutaneous nephrolithotomy without any other intervention was successfully utilized for the treatment of this stone.

Staghorn calculi (SC) are defined as large kidney stones that fill the renal pelvis and at least one renal calyx. They are typically radiopaque and composed of struvite; it is worth noting that struvite stones only form under conditions of increased ammonia production, which results in elevated urine pH^1^. Therefore, SC are usually associated with chronic urinary tract infections, alkaline urine, and urease-producing bacteria. Struvite stones account for up to 30% of urinary tract stones worldwide^2^. Particularly, struvite stones represent 10–20% of the urinary stone burden in developing countries, but only 4% of the burden in developed countries, due to improved diagnosis and management^3,4^. While the exact prevalence of struvite stones in the Middle East is unknown, it is estimated that the general occurrence of urolithiasis is higher in this region as compared to rest of the world – likely due to the combination of several risk factors unique to the area, such as hot and dry weather, dietary habits, and obesity^5^. Struvite stones are also twice as common in women than in men, despite the prevalence of renal stones being higher in men overall^4^. Most SC are unilateral, with only 15% of cases being bilateral^6^.

Other known risk factors for SC include female gender, neurogenic bladder, indwelling Foley catheters, or urinary diversions^7^. Due to their significant morbidity and mortality, timely diagnosis and management of SC is crucial. While large renal stones are usually managed via percutaneous nephrolithotomy (PCNL), it is recommended that bilateral SC or stones of unusual size be managed exclusively via surgical intervention – namely open, laparoscopic, or robotic surgery^8,9^. Additionally, conservative treatment of staghorn stones has been shown to carry higher mortality^10^. Herein, we report a case of a giant staghorn stone sized over 7.0 cm×5.0 cm, which was successfully managed via ultrasound-guided PCNL. Additionally, this staghorn stone was found to be compressing the inferior vena cava (IVC), possibly causing associated hepatomegaly – the first to be reported in the literature. This case report is reported in line with the SCARE (Surgical CAse REport) criteria^11^.

## Case presentation

### History and physical examination

A 63-year-old Saudi male presented to our clinic with a complaint of chronic abdominal pain and new-onset flank pain. The patient was alert, oriented, and vitally stable. A physical examination was conducted, and hepatosplenomegaly was noted; however, no ascites or jaundice were identified. Mild tenderness was noted in the right upper quadrant, along with slight lower extremity edema. All other physical examination findings were unremarkable. The patient was admitted, and intravenously (i.v.) paracetamol was administered.

### Investigations and findings

**Figure 1:**
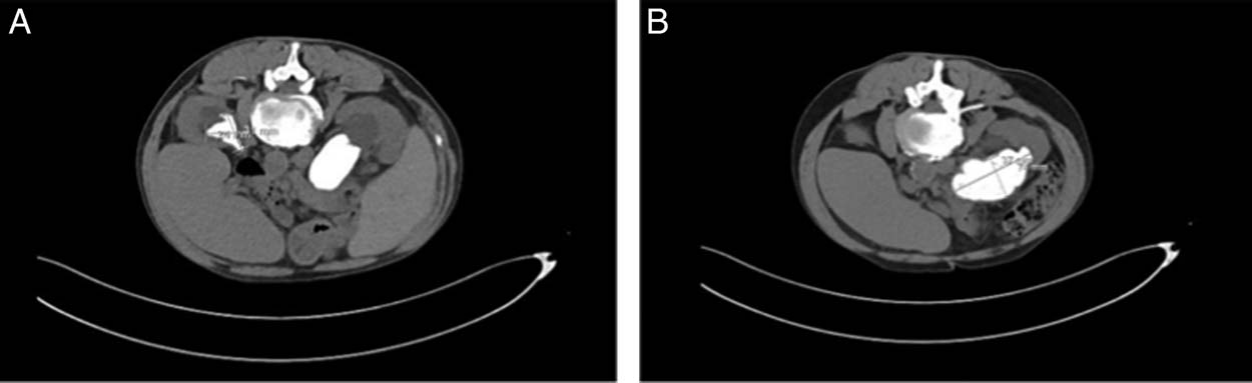
Axial view computed tomography scan taken upon initial presentation.

**Figure 2:**
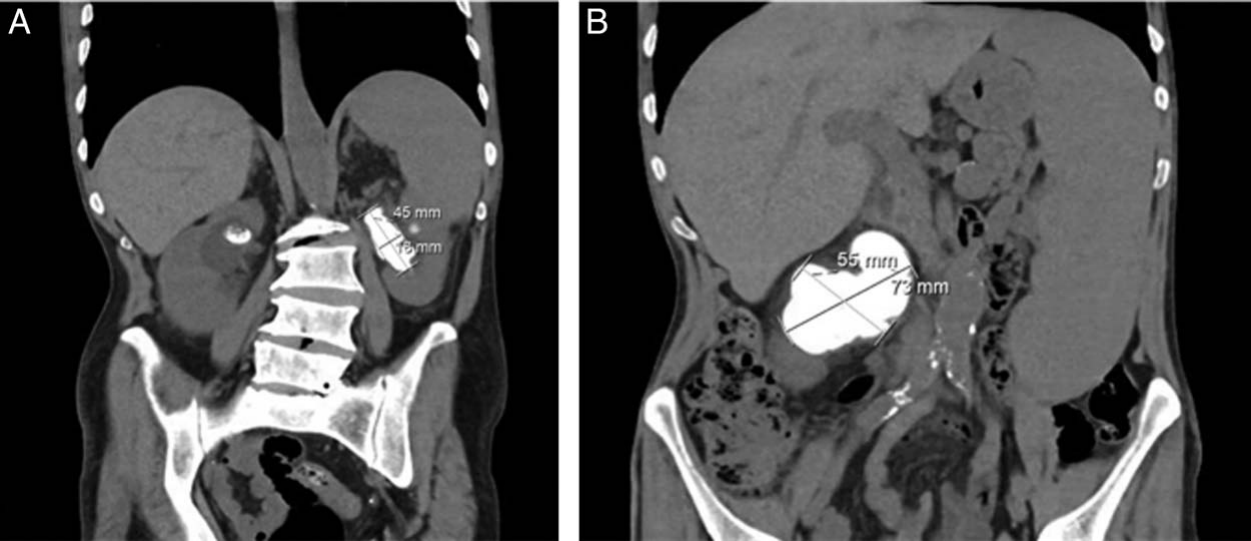
Coronal view computed tomography scan showing two large staghorn calculi, with compression of the inferior vena cava on the right.

Renal and liver function tests performed were normal. Kidney function and urine output were regular. A complete blood count revealed an elevated hemoglobin of 18.4 g/dl (normal: 13.5–16.5 g/dl), an elevated hematocrit of 57% (normal <49%), and increased red cell mass at least 25% greater than the mean normal predictive value^12^. After a comprehensive workup, a *JAK2*-positive diagnosis of polycythemia vera was made. Consequently, the patient was started on hydroxyurea, followed by venous thromboembolism prophylaxis. A noncontrast computed tomography (CT) scan of the abdomen was performed, and bilateral staghorn kidney stones were identified, measuring 7.3×5.5 cm on the right (occupying the right renal pelvis and calyces) and 1.8×4.5 cm on the left (as seen in Figs 1, 2, respectively). The right stone was noted to be compressing the IVC (Fig. 2). Hepatosplenomegaly was also noted. The patient was started on aspirin and i.v. fluids and was scheduled for right PCNL the following day.

### Treatment

**Figure 3:**
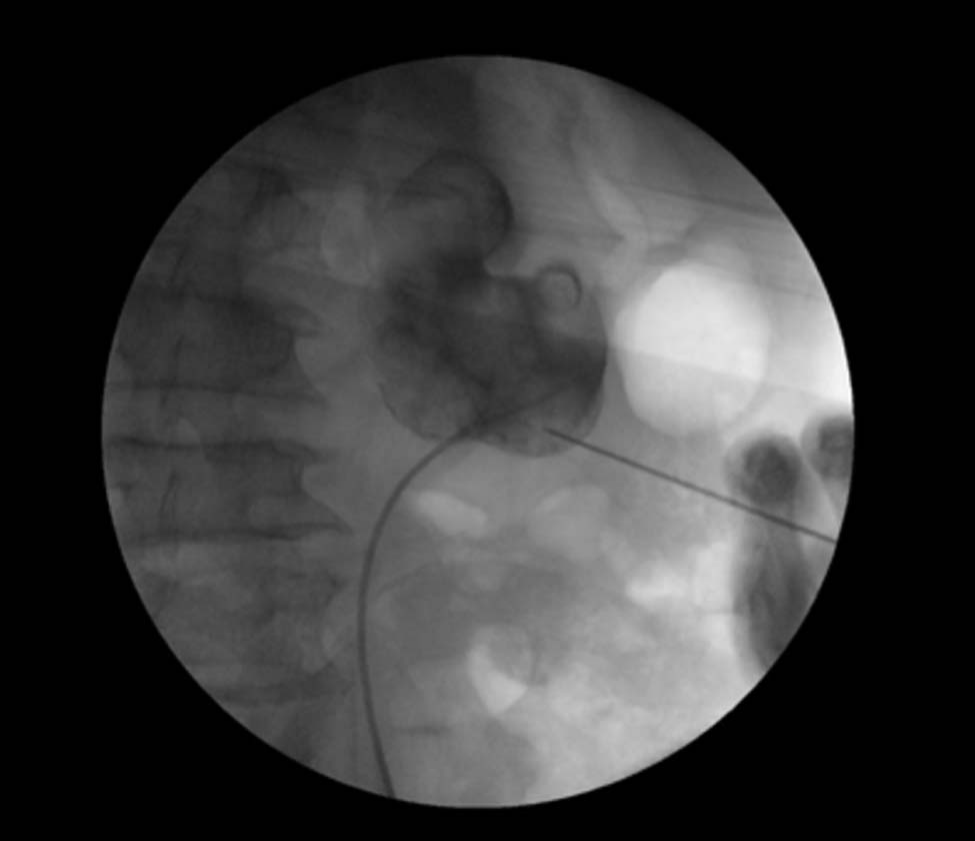
Lower pole subcostal access puncture site.

Intraoperatively, the patient was placed in a prone position under general anesthesia. A flexible cystoscope was inserted through the urethra, and the right ureteric orifice was identified. This was instrumented using a solo guidewire, and the coiling around the renal pelvis was visualized. After this, a 5-French (Fr) ureteric catheter was advanced over. Then, a 16-Fr Foley catheter was inserted, and both the Foley and ureteric catheters were secured. The stones were localized after injecting contrast material, and the guidewire was advanced. The puncture site was at the lower pole subcostal accesses (seen in Fig. 3). A hockey-stick ultrasound probe was advanced to the bladder, and the first guidewire was exchanged for an extra stiff guidewire. Next, the hockey-stick probe was lifted, and an 8×10 Fr dilator was advanced over the new guidewire. A safety guidewire was then advanced, and the balloon was dilated over the extra stiff guidewire using a 30-Fr access sheath. Fragmentation was initiated using the shock pulse machine and the combined ultrasonic and pneumatic lithotripter. The procedure was terminated when an estimated 80% of the stones had been fragmented. No bleeding from the access site or the nephrostomy tube was observed following the procedure.

### Follow-up and outcome

The patient was followed up for 6 months postoperatively. Within 4 weeks, the patient had excreted the left renal stone in their urine. A repeat ultrasound performed at 1 month revealed that normal kidney shape and size had been restored. Re-evaluation determined that the hepatosplenomegaly resolved spontaneously after 1.5 months. The patient is currently continued on hydroxyurea and aspirin.

## Discussion

SC are classified as upper urinary tract stones that occur in the renal pelvis extending to one or more calyces^4^. The prevalence of nephrolithiasis worldwide has risen, posing an exceedingly significant financial and clinical burden. While women are more likely to form struvite stones than men, Malik *et al*.^5^ identified that Saudi males are 8 times more likely to develop urolithiasis than Saudi females as a result of hormonal factors.

Due to the high risk of developing a superimposed infection, staghorn stones require prompt diagnosis and management. The gold standard treatment for massive SC is surgical; however, nonsurgical options may be added^10^. Conservative management is not recommended, as it has shown a 28% 10-year mortality rate and a 36% risk of progression to significant renal impairment^10^.

Very large SC have complex morphology, high recurrence rates, and infection-related complications. However, due to advancements in minimally invasive management strategies like shockwave lithotripsy, PCNL, retrograde ureteroscopy, and laparoscopy, several treatment options have recently emerged for the treatment of very large SC^13^.

In this article, we present the case of a 63-year-old suffering from polycythemia vera who presented to the clinic with chronic abdominal and flank pain. Upon physical examination, hepatosplenomegaly, mild ankle edema, and abdominal tenderness were noted. A noncontrast CT scan revealed bilateral SC. The right stone was found to measure 7.3×5.5 cm and appeared to be compressing the IVC, while the left stone measured 1.8×4.5 cm. The patient then successfully underwent right-sided PCNL with no residual bleeding from the access site or the nephrostomy tube later.

To date, the largest renal stone ever described is one measuring 13 cm, which was successfully removed via transperitoneal laparoscopic nephrectomy^14^. While the larger of the two stones reported in our case is only 7.3×5.5 cm in comparison, to our knowledge, it is the first of such incredible size to be reported in the region. Considering its impressive dimensions and the CT findings, it is evident that this stone was compressing the IVC – a finding that has not been reported previously. However, it is unclear whether the IVC compression (as caused by the large stone) was also responsible for the hepatosplenomegaly seen in this case.

On the one hand, kidneys are retroperitoneal organs, with the right renal vein directly draining into the IVC. In theory, a large staghorn stone located in the right renal calyces may expand to apply extrinsic pressure on the IVC, eventually reducing blood flow, causing backward congestion of the liver sinusoids and splenic blood vessels, and ultimately hepatosplenomegaly^15^. On the other hand, the enlarged liver and spleen seen in this patient may also be the result of increased cell turnover, as caused by polycythemia vera. Budd–Chiari syndrome (BCS) – a disorder that occurs due to hepatic vein and/or IVC obstruction secondary to a chronic myeloproliferative neoplasm (e.g. polycythemia vera) – is another potential cause. Budd–Chiari typically presents with three findings: abdominal pain, tender hepatomegaly, and ascites. While our patient only fits two of these, it appears that *JAK2* mutations are, in fact, detected in 35–50% of patients with Budd–Chiari, making it a credible diagnosis^16^. Importantly, however, is that the baseline renal function tests and liver function tests were also normal. It is unclear to what extent the patient’s hepatosplenomegaly may have been caused by IVC compression. Previous cases of IVC compression in literature list thrombus formation, tumor extension, and extrinsic compression as other causes of IVC compression^17^. Additionally, the symptoms reported in these cases include ascites, anasarca, and swelling of the lower limbs, while our patient only presented with slight lower limb edema – another unusual aspect of this case^18^. While the exact cause of hepatosplenomegaly is undetermined, significant conclusions may be drawn from this case to aid in future research. Greater studies are required on the possibility of SC causing IVC obstruction.

Several authors have recommended an invasive surgical approach for the management of massive SC of such proportions^10,19–21^. Surprisingly, however, our patient was successfully treated with only PCNL. While such a case regarding a stone of this size that was treated by PCNL only – particularly one compressing the IVC – has not been reported, Gavande and Gavande^22^ discuss a case of a 10 cm×7.5 cm stone, also successfully managed via PNCL. However, the authors admit to performing calyceal puncture, which was evaded in our patient. Greater research is needed on the potential utility of using ultrasound-guided PCNL for the fragmentation of stones sized over 5 cm.

## Conclusion

SC are growing to become exceedingly common, causing great financial and economic strain. In this case report, we present a unique case of IVC compression secondary to a massive staghorn stone seen in a 63-year-old male with polycythemia vera. To our knowledge, this is the first stone of such size to be reported in the region, as well as the first to be discussed in the context of IVC compression and hepatosplenomegaly. We hope this report elucidates the role of minimally invasive surgical procedures in the fragmentation of massive SC, as well as the association between SC and IVC compression. We highlight that ultrasound-guided PNCL without any other intervention is sufficient for the treatment of such stones, suggesting this strategy may be applicable to other massive stones.

## Ethical approval

Ethical approval is exempted in the case of a case report as per the King Faisal Specialist Hospital and Research Centre.

## Consent for publication

Written informed consent was obtained from the patient for the publication of this case report and accompanying images. Patient anonymity is maintained throughout this manuscript. A copy of the written consent is available for review by the Editor-in-Chief of this journal on request.

## Sources of funding

This study did not receive funding from any source.

## Author contribution

M.A.A., S.A.R., A.A., and M.T.A.: conceptualization; M.A.A., A.A., and T.A.: supervision; S.A.R., H.M.R., B.N.S., A.E.M., M.I.E.S., and A.E.A.: writing – original draft; M.A.A., S.A.R., B.N.S., A.E.M., M.I.E., A.E.A., A.A., T.A., M.T.A., and M.A.: writing – review and editing. All authors reviewed the manuscript for intellectual content and approved the submission.

## Conflicts of interest disclosure

The authors declare no conflicts of interest.

## Guarantor

Mohammad A. Alghafees, MD.

## Provenance and peer review

Not commissioned, externally peer-reviewed.

## Acknowledgments

None.
